# Identifying Gut Microbiota associated with Gastrointestinal Symptoms upon Roux-en-Y Gastric Bypass

**DOI:** 10.1007/s11695-023-06610-6

**Published:** 2023-04-24

**Authors:** Madelief Wijdeveld, Nienke van Olst, Eduard W. J. van der Vossen, Maurits de Brauw, Yair I. Z. Acherman, Marcus C. de Goffau, Victor E. A. Gerdes, Max Nieuwdorp

**Affiliations:** 1 Department of Internal and Vascular Medicine, Amsterdam University Medical Centers, Location AMC, Meibergdreef 9, Room D3-211, 1105 AZ Amsterdam, The Netherlands; 2 Department of Bariatric Surgery, Spaarne Gasthuis, Spaarnepoort 1, 2134 Hoofddorp, The Netherlands; 3 Tytgat Institute for Liver and Intestinal Research, Amsterdam University Medical Centers, Meibergdreef 69, 1105 BK Amsterdam, The Netherlands; 4 Sanger Institute, Saffron Walden, Cambridge, CB10 1RQ UK

**Keywords:** Roux-en-Y gastric bypass, Gastrointestinal Microbiome, Microbiota, Signs and symptoms, Digestive, Short-chain fatty acids

## Abstract

**Abstract:**

**Purpose:**

Roux-en-Y gastric bypasses (RYGB) are frequently accompanied by long-term gastrointestinal (GI) symptoms. Direct mechanistic insight into the causation of these symptoms is lacking, but changes in the intestinal microbiome have been proposed to play a role. With this study, we aimed to investigate whether a microbial predisposition exists before RYGB which is associated with GI symptoms during follow-up and to evaluate which microbial groups are involved.

**Materials and Methods:**

In total, 67 RYGB patients were included. Shotgun metagenomic sequencing was performed on fecal samples obtained just before and 1 year after surgery. To assess GI symptoms, patients filled out Gastrointestinal Quality of Life Index (GIQLI) questionnaires and were divided into groups based on their total GIQLI score and change in score (postsurgery versus baseline). Extremely randomized tree predictor models were used to identify the most distinctive microbial species associated with postoperative GI symptoms.

**Results:**

Beta diversity differed significantly between baseline and 1-year post-surgery samples, with the post-surgery microbiome resembling a more dysbiotic profile. The most predictive species regarding total GIQLI (AUC 0.77) or delta GIQLI score (AUC 0.83) were identified. Many of these species are known butyrate producers or species known to support them and/or species with anti-inflammatory properties, including *Coprococcus eutactus*, *Faecalibacterium prausnitzii*, and *Ruminococcus callidus*.

**Conclusion:**

Beneficial commensal gut microbiota related to a high GI score were associated to adequate intestinal fermentative capacity, suggesting these species might have protective properties against postoperative GI malfunctioning.

**Graphical Abstract:**

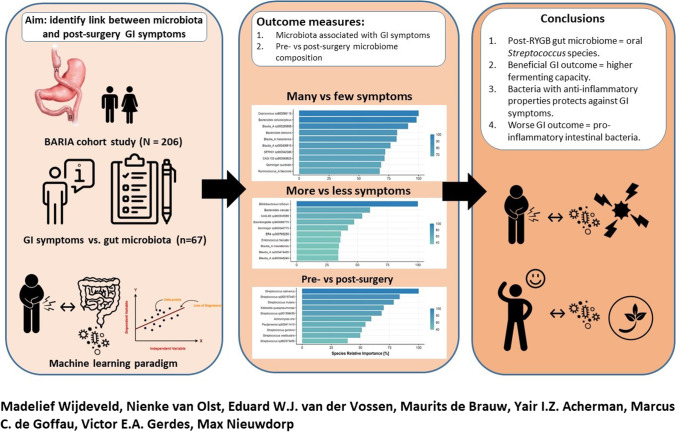

**Supplementary Information:**

The online version contains supplementary material available at 10.1007/s11695-023-06610-6.

## Introduction

The use of the Roux-en-Y gastric bypass (RYGB) procedure has now increased to 113,000 cases per year worldwide [1]. However, with the increased number of surgeries being performed, new problems are coming to light. An important problem is that RYGB is accompanied by long-term gastrointestinal (GI) symptoms, in nearly 50% of RYGB cases [2, 3]. Gastrointestinal symptoms after RYGB include chronic diarrhea, cramps, bloating, and bowel urgency and commonly lead to a decreased quality of life, limited general daily functioning, and increased absenteeism [4].

Relatively little is known about the etiology underlying the development of chronic GI symptoms after RYGB surgery. Given the lack of insight in regard to the underlying causes, doctors continue to experience difficulties in treating GI symptoms in gastric bypass patients. It has been suggested that the altered intestinal microbiota after RYGB could be a cause for these GI complaints as it undergoes significant changes after surgery [5, 6]. Various microbial changes are reported after RYGB; for instance, increases of various *Fusobacteria* and *Streptococcus* subspecies have been reported [7], whereas *Bifidobacterium* and *Firmicutes* are reported to decrease after surgery [8]. These changes result from an altered anatomy of the upper GI tract, increased stomach pH levels, altered carbohydrate, protein and bile acid metabolism, and an altered dietary pattern of the host [9]. Despite efforts, direct mechanistic insights into the causation of GI symptoms based on clinical data are mostly lacking. Westerink and colleagues unveiled certain inflammatory markers associated with GI symptoms after RYGB in a longitudinal bariatric cohort [10]. Microbiota data were, however, not reported in previous studies. A direct link between microbial compositional changes and GI symptoms has never been studied.

We therefore included 67 gastric bypass patients from a bariatric cohort (the BARIA study [11]) and applied various machine learning models to answer the question whether a relationship between microbiota changes and GI symptoms after surgery is present. Models were designed to predict the degree of GI complaints based on the intestinal microbiota composition. We hypothesized that increased GI symptoms after surgery are associated with the gut microbiota composition before surgery and that shifts in microbiota composition after surgery may also contribute to GI symptoms. We in particular predicted that oral microbiota strains, now more easily able to pass the gastric pouch alive and enter the lower intestinal system, would be increased in abundance after surgery and would be associated with GI symptoms. A connection between certain bacterial species and the severity of GI symptoms could potentially provide useful insights for postoperative treatment options of such symptoms, including dietary advice or pre- and probiotics.

## Research Design and Methods

### Study Design and Population

Included patients were participants from the BARIA cohort study [11]. In brief, the aim of this study is to identify novel pathways in the pathogenesis of obesity and obesity-related comorbidities through gut microbial, immunological, and metabolic markers in a large bariatric surgery cohort. All participants met the International Federation for the Surgery of Obesity and Metabolic guidelines of 2019: preoperative body mass index (BMI) ≥ 40 kg/m^2^ or BMI ≥ 35 kg/m^2^ with at least one associated medical condition and age of 18–65. All patients in this study underwent RYGB with a biliary limb length of 50 cm and alimentary limb of 150 cm. All steps of the study design including preoperative screening and procedural details of the operation are extensively reported in the published design of the BARIA Longitudinal Cohort Study [11]. Patients in the BARIA study undergo repetitive measurements within a follow-up of ten years. Two of the measurements consist of plasma and fecal sample collection, before surgery and one year after surgery. Furthermore, in the context of outpatient clinical care in the Spaarne Gasthuis Hoofddorp, all patients were asked to complete the Gastrointestinal Quality of Life Index (GIQLI) questionnaire before and annually after surgery. All subjects from the BARIA cohort who had completed their 2-year follow-up at time of screening and filled out the GIQLI questionnaires were approached for this study. In total, 67 participants were included. Study protocols were approved by the Ethical Review Board of the Academic Medical Center, Amsterdam (approval code: NL55755.018.15), and all patients provided written informed consent. All study procedures were in accordance with the declaration of Helsinki.

### Questionnaires

In order to measure GI symptoms in relation to RYGB, the commonly used and validated GIQLI Questionnaire was used [12]. This questionnaire consists of 36 questions from 5 different domains: core symptoms, disease-specific items, physical items, psychological items, and social items. A participant could score 0–4 points per question, and a total score of 0–144 points across the entire questionnaire. The lower the GIQLI score, the worse the GI quality of life. Based on previous research, the cutoff for a low GIQLI score in this study was 126 points [13, 14]. A total score of ≥ 126 was considered a high GIQLI score. Subsequently, the preoperative score was subtracted from the postoperative score to assess the delta in GIQLI score. A person with a delta of 0 or higher was considered having less symptoms, whereas someone with a negative delta was considered having more symptoms after surgery. Finally, the assessment of diarrhea or obstipation was performed by specific questions from the GIQLI questionnaire (2 regarding diarrhea, 1 regarding obstipation). The cut-off to consider a patient having diarrhea or obstipation was ≤ 4 and ≤ 2 points respectively.

In addition, all participants filled out an online nutritional diary (https://mijn.voedingscentrum.nl) to monitor daily caloric intake during the 3 days prior to every study visit in order to calculate daily average amount of consumed carbohydrates, fat, protein, and fiber.

### Gut Microbiota Sampling Methods

Participants were instructed to collect a fecal sample on the day of surgery (baseline) and on the day of the 1-year follow-up visit. The complete collection procedure is described elsewhere [15]. In brief, subjects were given a stool collection tube for transport and were asked to bring the sample to the study site within 6 hours after production and collection. If the sample was collected in the evening, subjects were instructed to keep the stool sample in their −20°C freezer overnight and to bring it on frozen icepacks to the research unit the next morning. Fecal samples were stored on site at −80°C.

Fecal total genomic DNA isolation was performed as previously described [16]. Genomic DNA was extracted from fecal samples by bead beating using a modified version of the IHMS DNA extraction protocol Q as described previously [17]. Fecal samples were dispersed in Lysing Matrix E tubes (MP Biomedicals) containing ASL buffer (Qiagen). Lysis was obtained after homogenization by vortexing for 2 minutes, by two cycles of heating at 90°C for 10 minutes followed by 3 bursts of bead beating at 5.5 m s^−1^ for 60 seconds in a FastPrep-24 instrument (MP Biomedicals). After each bead-beating burst, samples were placed on ice for 5 minutes. The supernatant containing the fecal DNA was collected after each bead-beating cycle by centrifuging at 4°C. Supernatants from the two centrifugation steps were pooled, and a 600-μL aliquot from each sample was purified using the QIAamp DNA Mini kit (QIAGEN) in the QIAcube instrument (QIAGEN) using the procedure for human DNA analysis. Samples were eluted in 200 μL of AE buffer (10 mM Tris-Cl, 0.5 mM EDTA, pH 9.0).

### Gut Microbiota Sequencing Methods

Libraries for shotgun metagenomic sequencing were prepared by a PCR-free method; library preparation and sequencing were performed at Novogene (Cambridge, UK) on a HiSeq instrument (Illumina) with 150-bp paired-end reads and 6 G data per sample.

### Fecal Microbiome Composition Analysis

The sequence classification algorithm of the Kraken pipeline [18] was used for preprocessing of raw shotgun metagenomics sequence data. Kraken is an ultrafast and highly accurate program for assigning taxonomic labels to metagenomic DNA sequences, which maps reads to reference databases, combines output from several sequencing runs, and manipulates tables of read counts. Quality-filtered reads were mapped to a genome catalog and gene catalog. After sequencing, raw reads were quality filtered including the removal of human reads using the Kneaddata tool (v. 0.10.0) with default settings (https://bitbucket.org/biobakery/kneaddata). Taxonomy was assigned on pairs of high-quality reads using Kraken2 with the paired option [19] and the UHGG database v. 2.0 [20]. Bracken v. 2.6.2 was used throughout for species level classification [21]. The resulting data from these pipelines were processed using R (v. 4.0.2) in the RStudio IDE (v. 1.3.1093). Alpha diversity indices were computed and visualized using the phyloseq (v. 1.34.0) [22], vegan (v. 2.5), and ggplot2 [23] packages, and beta diversity was computed using Bray Curtis dissimilarity distances between samples.

### Statistical Analysis

This study concerns two primary outcomes. First, the relation between microbial composition (pre- and postsurgery) and postsurgery GIQLI score. Second, the relation between microbial composition and the change in GIQLI score upon surgery. As secondary outcome, a logistic regression was performed between the 10 most predictive species (based on machine learning) and dichotomized postoperative GIQLI score, corrected for baseline GIQLI score. Furthermore, linear regression was performed between the decrease in GIQLI score upon surgery in patients with a delta GIQLI of ≤ −10 (*N* = 21) and the 10 species that were most predictive for a decrease in GIQLI score postsurgery.

#### Univariate Analyses

Unless stated otherwise, R Studio v. 4.0.3 was used to perform all statistical analyses described below. Differences in clinical variables were compared between the baseline and the 1-year follow-up visit. Furthermore, clinical characteristics of participants’ 1-year postsurgery were compared between the “high” and the “low GIQLI score” group and the “more” versus the “less symptoms” group. Data were tested with paired or unpaired *T*-test or Mann-Whitney *U* test, depending on Gaussian distribution. Pearson’s chi-square test was used to test for differences in sex and medication use between both groups. A *p*-value < 0.05 was considered significant.

#### Machine Learning

An extremely randomized tree machine learning algorithm was applied to predict the class of a subject based on their baseline and one year follow-up microbiome data. Machine learning was implemented in Python (v. 3.8.5) using numpy (v. 1.16.4), pandas (v. 0.25.1), and scikit-learn (v. 0.21.2) packages. Three separate classification models were run. The first model was used to differentiate between baseline and 1 year postsurgery microbiota composition. The second model was applied to identify a panel of gut microbiota that best predicted the dichotomized postoperative GIQLI scores, thus indicating whether a subject belonged to the “high” or “low GIQLI score” group. A final third model was run to identify a panel of gut microbiota that best predicted whether a subject belonged to the “more symptoms” or “less symptoms” group after surgery. Gut microbiota were filtered prior to each simulation to reduce dimensionality. To find the microbiota that play a potential larger role, an abundance filter was applied to select the top 200 most abundant species among all patients. Hereafter, a univariate feature selection was applied (SelectPercentile) to select the 50 features that were subsequently used in each simulation. The models were used in a nested cross-validation structure to prevent overfitting and to ensure robustness of results. The LeaveOneOut Cross-Validation method was applied, meaning that all samples except for one were used in the training set, and the one sample left out was used for testing. For hyperparameter tuning, a three-fold stratified shuffle split was used in which 80% was trained and 20% was cross-validated. The optimized model was then tested on the examples in the test set. The permutation feature importance was extracted from the model.

## Results

### Clinical Characteristics and GIQLI Score

**Fig. 1:**
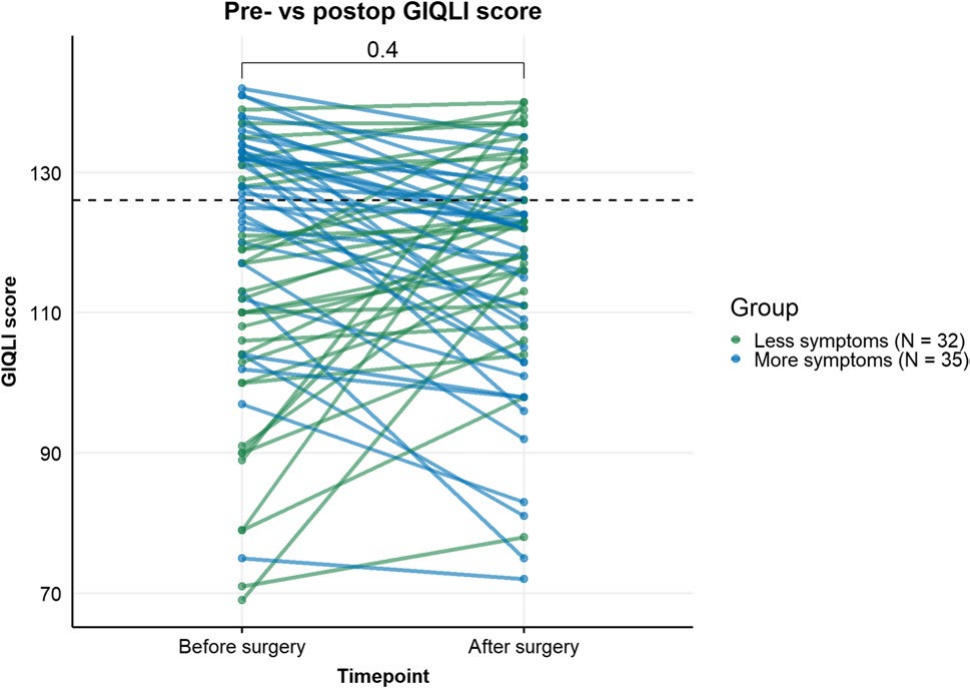
Baseline versus 1-year postsurgery GIQLI score. Cut-off for a “high” versus “low GIQLI score” was 126 points. Green: people with less symptoms after surgery compared to baseline. Blue: people with more symptoms after surgery compared to baseline

In total, 67 participants were included in this study. All included patients underwent RYGB. Clinical characteristics at baseline and at the 1-year follow-up are presented in Table 1. One year after the RYGB surgery, as expected, a significant decrease in body weight, BMI, fat percentage, and metabolic parameters was observed (*p* < 0.001). The median GIQLI score was 121 [105–132] points before surgery and 122 [108–128] points after surgery. Pre- versus postoperative GIQLI scores are depicted in Fig. 1. Finally, the groups did not differ in baseline macronutrient intake based on the food diaries (data not shown). Similarly at 1-year post-surgery no differences were found between the GIQLI groups in dietary intake.

**Table 1:** Characteristics of study subjects (*N* = 67)

	Baseline visit (presurgery)	1-year postsurgery	*p*-value
Age	47.88 (8.92)	48.88 (8.92)	**<0.001**
Female, *n*= (%)	51 (76.1%)	51 (76.1%)	1.000
BMI	**39.92 (4.13)**	**30.61 (3.83)**	**<0.001**
Body weight	**127.22 (16.75)**	**91.54 (12.92)**	**<0.001**
Body fat (%)	**46.0 (4.98)**	**29.27 (6.77)**	**<0.001**
Systolic blood pressure (mmHg)	**133.8 (14.2)**	**122.3 (13.4)**	**<0.001**
Diastolic blood pressure (mmHg)	**82.0 (9.8)**	**81.0 (7.8)**	**<0.001**
Heart rate (bpm)	75.4 (12.0)	74.7 (13.7)	0.8507
Total cholesterol (mmol/L)	**4.7 [4.1–5.6]**	**4.4 [3.8–4.7]**	**<0.001**
HDLc (mmol/L)	**1.2 [1.0–1.395]**	**1.5 [1.3–1.7]**	**<0.001**
LDLc (mmol/L)	**3.0 [2.4–3.8]**	**2.5 [1.9–2.9]**	**<0.001**
Triglyceride (mmol/L)	**1.4 [1.1–1.7]**	**1.0 [0.7–1.3]**	**<0.001**
Medication use, *n*= (%)	46 (68.7%)	28 (41.8%)	0.0802
Metformin	13 (19.4%)	5 (7.5%)	**<0.001**
Proton pump inhibitor	20 (29.9%)	23 (34.3%)	**0.0058**
Anti-hypertensives	16 (23.9%)	11 (16.4%)	**<0.001**
Statins	12 (17.9%)	9 (13.4%)	**<0.001**

Baseline characteristics presurgery versus 1 year postsurgery, tested with paired *T*-test or Mann-Whitney *U* test, based on Gaussian distribution. Pearson’s chi-square test had been performed to test for differences in medication use between both groups. Numerical values are expressed as means ± standard deviations or median (IQR) depending on Gaussian distribution. Values in bold indicate significant difference between baseline and 1-year postsurgery visit, *p*-value < 0.05

*BMI* body mass index, *bpm* beats per minute, *HDLc* high-density lipoprotein cholesterol, *LDLc* low-density lipoprotein cholesterol, *TG* triglycerides

### Preoperative versus Postoperative Microbiota Composition

**Fig. 2:**
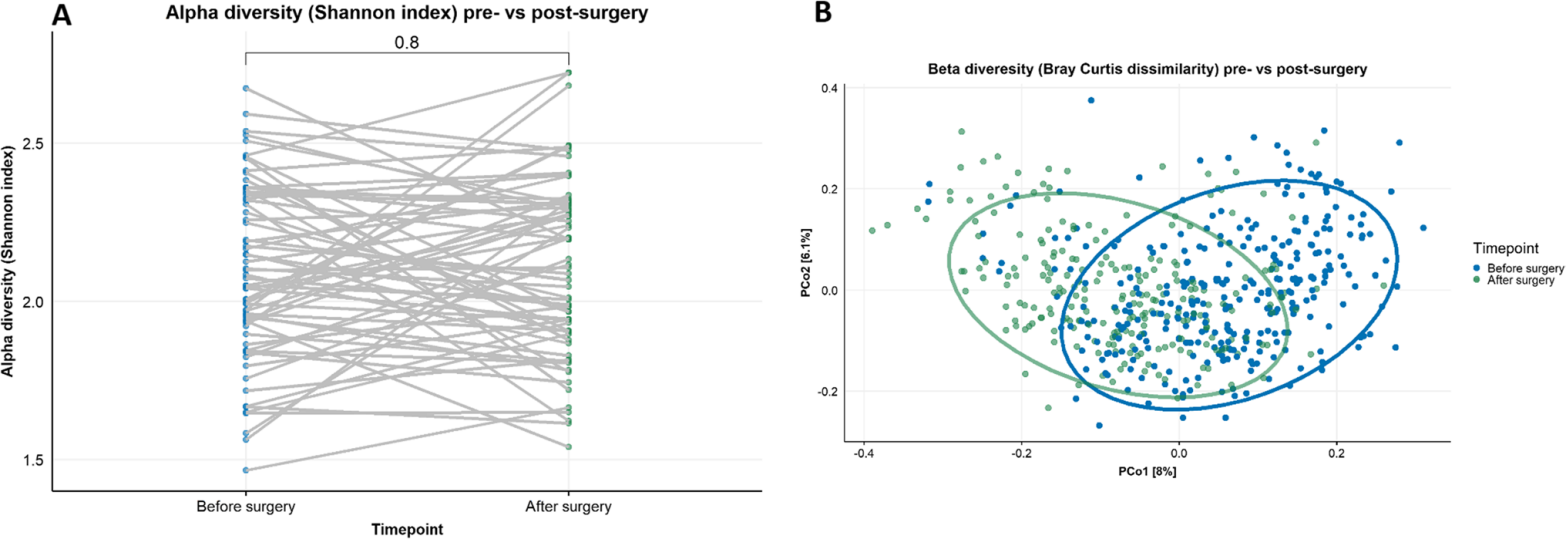
Alpha diversity pre- versus postsurgery. A Shannon index (blue: baseline samples, green: post-surgery samples). No difference in alpha diversity was observed. B Principal coordinate analysis (PCoA) on beta-diversity (Bray Curtis dissimilarity) between baseline and 1-year post-surgery fecal microbiota (PERMANOVA *p* = 0.0001, *R*^2^ = 3.15%, explained variance by first 2 PCo 8.0% and 6.1%). Each point represents one sample from one participant (blue: baseline samples, green: postsurgery samples). Closeness of points represents similarity of microbial composition

Changes in the gut microbiota after RYGB are presented in Fig. 2. No differences in alpha diversity were observed (Fig. 2a). However, the beta diversity (Bray Curtis) was significantly affected by surgery (PERMANOVA *p* = 0.0001, *R*^2^ = 3.15%, explained variance by first 2 PCo 8.0% and 6.1%) (Fig. 2b).

**Fig. 3:**
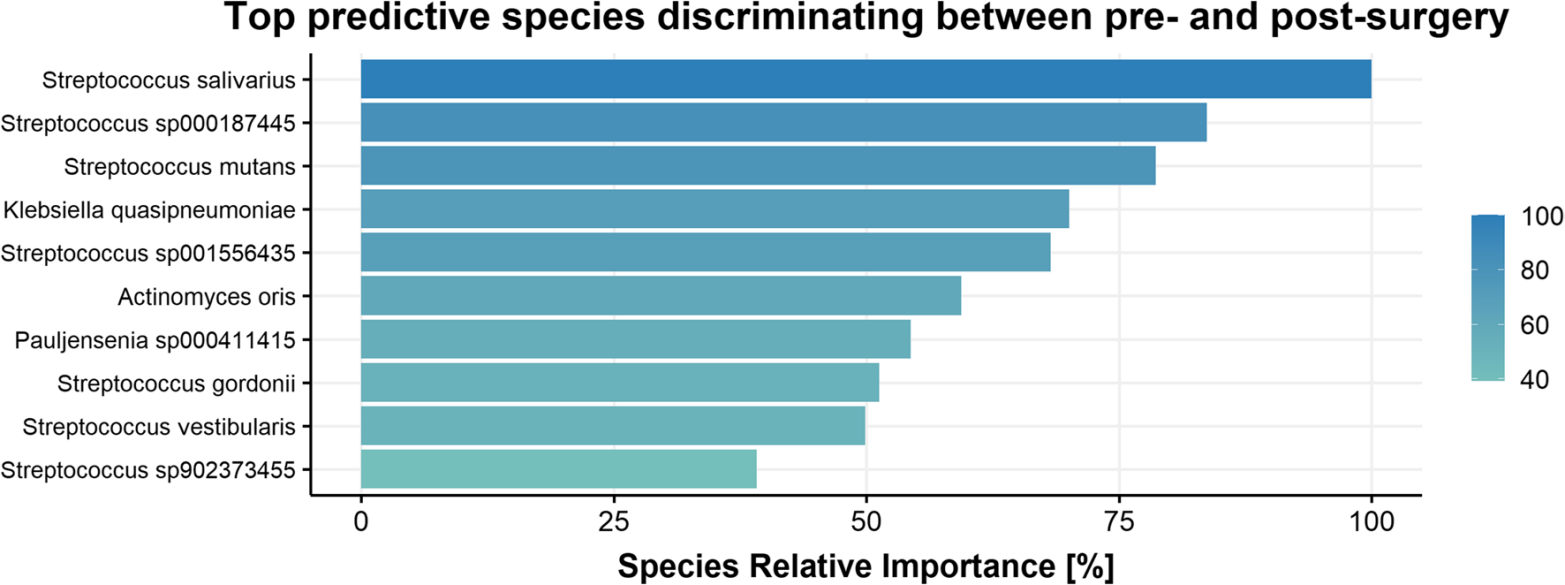
Relative feature importance of top 10 predictor species from gut microbiota composition for fecal microbiota composition pre- versus postsurgery. Relative importance is determined with respect to the most important predictor of the model, which was set to 100%. Predictor species were identified applying an extremely randomized trees classification model

RYGB surgery induced significant changes in the abundancies of several microbial groups. The machine learning model used to distinguish between the microbiota composition before and after surgery had an AUC of 0.95, indicating a very good discriminative ability between the pre- and postsurgery microbiota composition. Several *Streptococcus* (including *Streptococcus salivarius* and *Streptococcus mutans*) and various *Enterobacteriaceae* were significantly increased 1 year after surgery. See Fig. 3 for the depiction of top 10 most predictive species and supplementary Figure S1 for the top 4 most predictive species abundance per timepoint.

### Clinical Characteristics in Low versus High GIQLI Score Group

Table 2 shows clinical characteristics one year after the RYGB surgery in people with low versus high GIQLI scores (a score < 126 was considered low, ≥ 126 was considered high). No differences in clinical demographics were observed between the groups.

**Table 2:** Characteristics of subjects one year after surgery with low versus high GIQLI scores

	Low GIQLI score (*N* = 46)	High GIQLI score (*N* = 21)	*p*-value
Age	48.85 (8.85)	48.95 (9.30)	0.965
Female, *n*= (%)	37 (80.43%)	14 (66.67%)	0.359
BMI	28.20 (4.65)	27.25 (3.27)	0.399
Body fat (%)	30.21 (6.81)	27.13 (6.35)	0.103
Total weight loss (%)	34.76 (6.27)	33.60 (7.27)	0.533
Systolic blood pressure (mmHg)	121.85 (13.73)	123.19 (12.91)	0.707
Diastolic blood pressure (mmHg)	75.17 (7.68)	77.33 (7.99)	0.296
Heart rate (bpm)	75.91 (12.71)	72.19 (15.79)	0.307
Total cholesterol (mmol/L)	4.2 [3.6–4.7]	4.4 [3.9–4.8]	0.201
HDL cholesterol (mmol/L)	1.14 [1.10–1.60]	1.52 [1.45–1.91]	0.152
LDL cholesterol (mmol/L)	2.49 [1.79–2.98]	2.44 [2.1–2.75]	0.811
Triglyceride (mmol/L)	1.0 [0.71–1.45]	0.91 [0.64–1.12]	0.380
HbA1c (%)	5.34 (0.33)	5.42 (0.43)	0.409

Characteristics of BARIA study subjects postsurgery with high versus low GIQLI scores. A score < 126 points was considered low; a score ≥ 126 points was considered a high score. Data were tested with unpaired *T*-test or Mann-Whitney *U* test, based on Gaussian distribution. Pearson’s chi-square test had been performed to test for differences in sex and medication use between both groups. Numerical values are expressed as means ± standard deviations or median (IQR) depending on Gaussian distribution. No differences in characteristics were found between the groups

*HbA1c* hemoglobin A1c (glycated hemoglobin)

### Microbiota Composition in Low versus High GIQLI Score Group

**Fig. 4:**
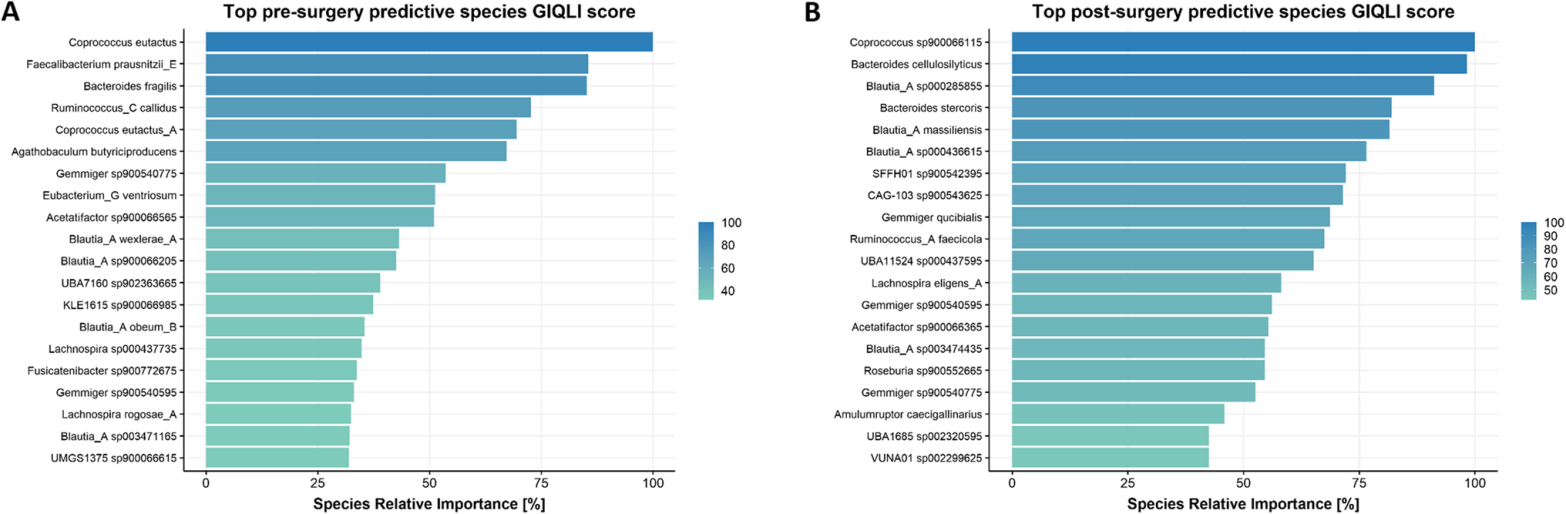
Relative feature importance of top 20 predictor species from gut microbiota composition for fecal microbiota composition predicting dichotomized GIQLI score (“low” versus “high GIQLI score”). All top species depicted were more enriched in the “high GIQLI score” group. Relative importance is determined with respect to the most important predictor of the model, which was set to 100%. Predictor species were identified applying an extremely randomized trees classification model. **A** Based on baseline microbiota composition. **B** Based on 1-year postsurgery microbiota composition

**Fig. 5:**
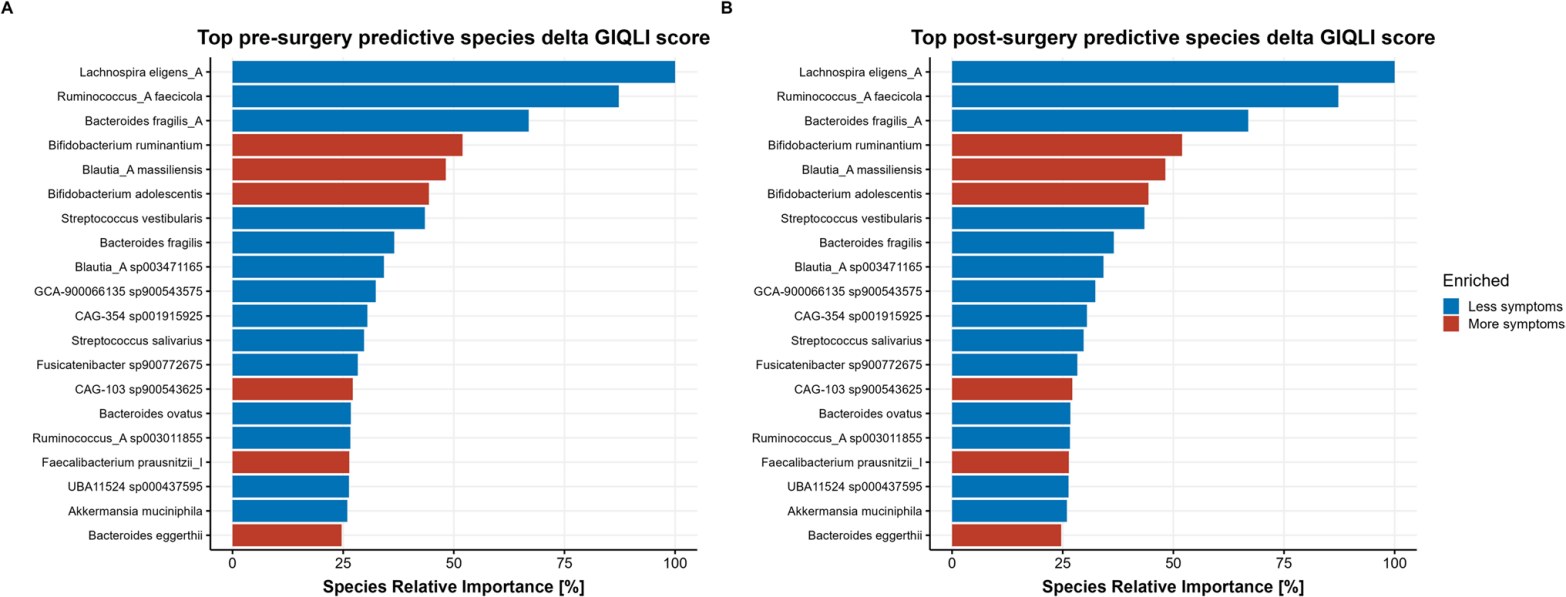
Relative feature importance of top 20 predictor species from gut microbiota composition for fecal microbiota composition predicting dichotomized GIQLI score (“more” versus “less GI symptoms”). Blue species are more enriched in the “less GI symptoms” groups; red are more enriched in the “more GI symptoms” group. Relative importance is determined with respect to the most important predictor of the model, which was set to 100%. Predictor species were identified applying an extremely randomized trees classification model. **A** Based on baseline microbiota composition. **B** Based on 1-year postsurgery microbiota composition

Lists of the top 20 most predictive microbial species which indicate whether a person belonged to the “low” or “high GIQLI score” group after surgery are depicted in Fig. 4a, and the abundances of the top 4 most predictive species are depicted in supplementary Figure S2. A high score appears to be associated with a higher preoperative relative abundance of species such as *Coprococcus eutactus*, *Faecalibacterium prausnitzii*, and *Bacteroides fragilis* (Fig. 5a)*.* Logistic regression revealed a significant fit between dichotomized postsurgery GIQLI score (corrected for baseline GIQLI score) and *Ruminococcus callidus, Agathobaculum butyriproducens*, *Eubacterium ventriosum*, and *Blautia wexlerae* abundance presurgery, all of which were more enriched in the “high GIQLI score” group.

In line, postoperatively increased abundance of *Bacteroides cellulosilyticus* and various *Blautia* subspecies, including *Blautia massiliensis*, appears to be associated to a high GIQLI score, meaning fewer GI symptoms (Fig. 4b). Again, the abundance of the top 4 most predictive species is depicted in supplementary Fig. S3. The pre- and postoperative machine learning model yielded an AUC of 0.77 and 0.69 respectively, indicating a moderate discriminative ability of the model in regards to the “low” and “high GIQLI score” group. Logistic regression revealed a significant fit between dichotomized post-surgery GIQLI score (corrected for baseline GIQLI score) and *Blautia* (resp. *massiliensis*, *sp000285855*, and *sp000436615*) and *Gemmiger qucibialis* abundance postsurgery, all of which were more enriched in the “high GIQLI score” group.

### Clinical Characteristics in More versus Less Gastrointestinal Symptoms Group

Table 3 shows the clinical characteristics one year after the RYGB surgery in people with more versus less GI symptoms as determined by their GIQLI score (a difference in score below 0 was considered more symptoms; an equal or increased score was considered less symptoms). No differences in clinical demographics were found between both groups.

**Table 3:** Characteristics of subjects postsurgery with more versus less GI symptoms

	More symptoms (*N* = 35)	Less symptoms (*N* = 32)	*p*-value
Age	46.80 (9.43)	49.06 (8.32)	0.303
Female, *n*= (%)	27 (77.14%)	24 (75.00%)	1.00
BMI	28.46 (4.87)	27.29 (3.45)	0.267
Body fat (%)	30.10 (7.28)	28.21 (6.03)	0.289
Total weight loss (%)	35.02 (6.37)	33.71 (6.81)	0.420
Systolic blood pressure (mmHg)	122.86 (14.82)	121.62 (11.84)	0.710
Diastolic blood pressure (mmHg)	76.34 (8.32)	75.31 (7.25)	0.592
Heart rate (bpm)	75.94 (13.60)	73.44 (13.99)	0.460
Total cholesterol (mmol/L)	4.20 (3.60-4.70)	4.40 (3.90-4.85)	0.097
HDL cholesterol (mmol/L)	1.45 (1.08-1.59)	1.5 (1.40-2.02)	0.647
LDL cholesterol (mmol/L)	2.49 (1.78-2.95)	2.44 (2.05-2.80)	0.576
Triglyceride (mmol/L)	0.88 (0.70-1.19)	1.1 (0.75-1.57)	0.124
HbA1c (%)	5.38 (0.44)	5.34 (0.27)	0.657

Characteristics of BARIA study subjects postsurgery with more versus less GI symptoms. A delta GIQLI score of < 0 was considered “more symptoms”; a score of ≥ 0 was considered “less symptoms”. Tested with unpaired *T*-test or Mann-Whitney *U* test, based on Gaussian distribution. Pearson’s chi-square test had been performed to test for differences in sex and medication use between both groups. Numerical values are expressed as means ± standard deviations or median (IQR) depending on Gaussian distribution. No differences in characteristics were found between the groups

### Microbiota Composition in More versus Less Gastrointestinal Symptoms Group

The top 20 most predictive species indicating whether a person belonged to the “more symptoms” or “less symptoms” group are depicted in Fig. 5, and the abundance of the top 4 is given in supplementary Figures S4 and S5. Less symptoms appear to be associated with higher preoperative abundances of species such as *Lachnospira eligens*, *Ruminococcus faecicola*, and *Bacteroides fragilis*, whereas *Bifidobacterium ruminantium* was more enriched in the “more symptoms” group (Fig. 5a)*.*

Postoperative increased abundances of *Bifidobacterium bifidum*, *Enterococcus faecalis*, and *Blautia massiliensis* appear to be associated with less symptoms, whereas *Bacteroides caccae* and *Paraprevotella clara* were more enriched in the “more symptoms” group (Fig. 5b). The pre- and postoperative machine learning model rendered an AUC of 0.83 and 0.78 respectively, indicative of a good distinctive ability between the “more” and “less symptoms” group.

Linear regression revealed no significant association between decrease in GIQLI score (in patients with a delta GIQLI of ≤ −10) and the abundance of any of the top predictor species depicted in Fig. 5.

## Discussion

In this trial, we studied the effects of changes in the gut microbiota composition on GI symptoms after RYGB. We related the pre- and postsurgery microbiota to postoperative GIQLI scores and the differences in GI symptoms pre- versus postsurgery. Hereby, we identified several pre- and postsurgery bacterial species of which the abundance predicted postoperative GIQLI score.

We observed a significant beta diversity difference when comparing pre- and postsurgery fecal microbiota compositions. Especially striking was the significant increase postsurgery of various *Streptococcus* species, including *Streptococcus salivarius* and *Streptococcus mutans*, which are generally mainly abundant in the oral cavity and upper GI tract [24, 25] and of oral *Prevotella* species. Furthermore, the postsurgery microbiome appeared to be fitting a more dysbiotic profile, characterized by more *Enterobacteriaceae* and a greater beta diversity within the postsurgery sample group. These findings suggest that RYGB induces a shift of the gut microbiota composition characterized by an increase in abundance of common upper GI tract bacteria. This increase might be caused by shorter transit time of bacteria through the gastric pouch, as well as the increased pH levels compared to the normal acidic stomach, leading to higher introduction rates of upper GI bacteria into the lower intestines [26, 27]. Stefura et al. observed an increase in abundance of the Bacteroidetes phylum, including Bacteroides and Odoribacter, in both the oral cavity and the large intestine 6 months after surgery among patients undergoing bariatric surgery [26]. In a previous publication reporting on a prospective cohort study, they found that specific compositions of the microbiota of the oral cavity and large intestine 6 months after bariatric surgery were associated with weight-loss [28]. However, to our knowledge, no study has established a direct relation between alterations in the intestinal microbiota composition and the oral cavity microbiota in RYGB patients.

Next, we examined which microbial species were most predictive for the postoperative GIQLI score. In the group classified as having a “high GIQLI score,” we characterized a distinct presurgery microbiota signature typified by a higher abundance of species such as *Coprococcus eutactus* and *Faecalibacterium prausnitzii*, suggesting that these commensal butyrate producing species might have protective properties against postoperative GI malfunctioning, possibly by enhancing intestinal barrier function [29, 30]. *Ruminococcus callidus* was also more abundant in people with a high GIQLI score. This species has previously been suggested to possess anti-inflammatory properties as it was found to be less abundant in patients with Parkinson’s disease [31]. Additionally, this fiber degrader is part of the same set of bacteria, including *F. prausnitzii*, that was found to be decreased in prediabetic children [32]. Possibly, a symbiotic interaction between *R. callidus* and *F. prausnitzii* could explain why these bacteria both appear to be protective. These findings combined suggest that increased baseline levels of the above-mentioned bacterial species could be beneficial in averting GI complaints after surgery. Postsurgery, we observed that the gut microbiota of people with high GIQLI scores encompassed more *Bacteroides cellulosilyticus*, which is capable of degrading various types of cellulose and sugars, hereby producing short-chain fatty acids and succinate [33]. Furthermore, various *Blautia* strains were more abundant in people with high GIQLI scores. RYGB commonly induces a decrease in *Blautia* abundance [34]; therefore, the retention of these commensals postoperatively may be associated with a better GI outcome.

With respect to the within-subject change in GI symptoms (delta GIQLI score), we found that species such as *Lachnospira eligens*, *Ruminococcus faecicola*, and *Bacteroides fragilis* were preoperatively more enriched in the participants in the “less symptoms” group. Of these species, *L. eligens* was suggested to have beneficial effects on intestinal health in an extensive clinical trial on the modulatory effects of the Mediterranean diet [35]. In this study, *L. eligens* was positively associated with several markers of lower frailty and increased short/branched chain fatty acid production, and correlated negatively with inflammatory markers such as IL-2 and C-reactive protein. *Bifidobacterium ruminantium* and *Bifidobacterium adolescentis* were more enriched in the “more symptoms” group*.*

In contrast, after surgery, an increase in *Bacteroides caccae* was observed in the “less symptoms” group. This is a so-called bidirectionally functional strain, able to metabolize both mucus polysaccharides and dietary fiber [36]. Certain *Eisenbergiella, Gemmiger*, and *Streptococcus* subspecies were more enriched in the “more symptoms” group. Intestinal bacterial overgrowth with *Streptococcus* has previously been observed after bariatric surgery [26, 37] and has been linked to abdominal infections [38].

Altogether, differences in GIQLI score were mostly explained by species such as *Ruminococcus callidus* and *Ruminococcus faecicola*, which have been suggested to be protective against inflammation since they were less abundant in patients with inflammatory bowel disease (IBD) and Parkinson’s disease [31, 39]. Furthermore, species associated with the fermentation of dietary fiber, such as *Coprococcus eutactus*, *Faecalibacterium prausnitzii*, and *Bacteroides cellulosilyticus*, may be favorable with regard to GI symptoms after surgery. It has to be noted that *F. prausnitzii* is not a very prominent digesting fiber by itself, but in the presence of *R. callidus*, the abundance of *F. prausnitzii* increases with the intake of dietary fiber [40]. Higher production rates of acetate, propionate, and butyrate, end products of anaerobic fermentation, may be indicative of a diminished accumulation of intermediate fermentation products associated with gas formation, specifically H_2_ and methane [41]. Finally, this study highlights that a preoperative microbial disposition may exist to develop more GI symptoms after RYGB.

Our current study comprised exploratory analyses of a pre- and postbariatric surgery population, regarding GI symptoms in relation to microbial composition. Its findings may contribute to future studies aimed at alleviating postoperative gastrointestinal discomfort. Based on our findings, dietary advice should be aimed at increasing the abundancy of *Ruminococcus* and *Faecalibacterium* species. Furthermore, pre- or probiotic supplementation can be considered to increase intestinal fermentative capacity [42], especially in patients with a presurgery microbiome composition associated with an increased risk of GI symptoms. Our findings suggest a predominant role of the microbiome in the development of GI symptoms after RYGB. However, no direct causal relationship can be established from this study. Furthermore, future studies with sufficient power should also look into possible alterations in bacterial metagenomic functional pathways and include antibiotic resistance as outcome measure. To our knowledge, this is the first study applying machine learning to assess which microbiota can be linked to GI symptoms after RYGB. This was done in a large and well-phenotyped bariatric surgery cohort, allowing the researchers to study the microbiome at multiple timepoints and examine the effect of the surgery. Finally, the microbiome data was analyzed using shotgun sequencing, providing a robust and reproducible method for bacterial composition analysis [43].

This study also has certain limitations. First, the microbiome composition is cumbersome to study after bariatric surgery, due to the altered ecosystem that is introduced, leading to more dysbiosis and a highly dispersed microbiome. We therefore also utilized the baseline microbiota data to identify a predisposition for development of symptoms postoperatively. Both prediction models rendered areas under the curve of 0.69 or higher, but did not show a high predictive value. However, it does show that the microbiota contributes to some extent to post-surgery symptoms, although it does not explain the entire variance of these symptoms. Furthermore, this study involved GI symptoms which can be considered multifactorial, therefore the exact contribution of the microbiome to the GIQLI score cannot be derived from this study and will always be relative to a multiplicity of underlying factors including diet, medication and lifestyle. Nevertheless, all these factors also affect the microbiome, and therefore, microbiome improvement can be considered a relevant component of perioperative treatment in RYGB patients.

With this study, we looked at the 1-year postoperative alterations in the microbiota after RYGB; we identified the most predictive species for postoperative GI symptoms score and for the postoperative increase or decrease of GI symptoms. Here, we identified predictors of either a beneficial GI score or a negative GI score. This distinction may be attributed to certain fermentative capacities or the production of either anti- or proinflammatory metabolites by the intestinal microbiota. Larger prospective cohort studies should include postbariatric surgery metabolomics profiles to confirm this.

## Supplementary information

ESM 1 (JPG 453 kb)

ESM 2 (JPG 301 kb)

ESM 3 (JPG 301 kb)

ESM 4 (JPG 310 kb)

ESM 5 (JPG 305 kb)

## Data Availability

Fecal metagenomic shotgun data have been deposited in the European Nucleotide Archive (ENA; PRJEB47902). Data from this particular clinical trial data can be requested by any qualified researchers who engage in rigorous, independent scientific research and will be provided following review and approval of a research proposal and statistical analysis plan and execution of a data sharing agreement by the corresponding author.

## References

[1] Ettinger J, Ázaro E, Weiner R (2020). Gastric bypass: bariatric and metabolic surgery perspectives.

[2] Boerlage T, Westerink F, Gerdes V (2017). Changes in gastrointestinal complaints and food intolerances after Roux-en-Y gastric bypass surgery. Surg Obes Relat Dis.

[3] Boerlage TCC, van de Laar AWJM, Westerlaken S (2017). Gastrointestinal symptoms and food intolerance 2 years after laparoscopic Roux-en-Y gastric bypass for morbid obesity. Br J Surg.

[4] Barbini N, Beretta G, Minnucci M (2006). Le principali patologie causa di assenza dal lavoro. Analisi della banca dati INPS [The main illnesses causing absence from work. Analysis of the INPS data bank]. G Ital Med Lav Ergon.

[5] Murphy EF, Quigley EMM (2012). The microbiota and bariatric surgery: it's a bug's life. Gastroenterology.

[6] Furet J-P, Kong L-C, Rizkalla S (2010). Differential adaptation of human gut microbiota to bariatric surgery-induced weight loss: links with metabolic and low-grade inflammation markers. Diabetes.

[7] Luijten JCHBM, Vugts G, Nieuwenhuijzen GAP (2019). The importance of the microbiome in bariatric surgery: a systematic review. Obes Surg.

[8] Li JV, Ashrafian H, Bueter M (2011). Metabolic surgery profoundly influences gut microbial–host metabolic cross-talk. Gut..

[9] Sanmiguel CP, Jacobs J, Gupta A (2017). Surgically induced changes in gut microbiome and hedonic eating as related to weight loss: preliminary findings in obese women undergoing bariatric surgery. Psychosom Med.

[10] Westerink F, Huibregtse I, De Hoog M (2021). Faecal inflammatory biomarkers and gastrointestinal symptoms after bariatric surgery: a longitudinal study. Inflamm Intest Dis.

[11] van Olden CC, van de Laar AW, Meijnikman AS (2020). A systems biology approach to understand gut microbiota and host metabolism in morbid obesity: design of the BARIA Longitudinal Cohort Study. J Intern Med.

[12] Eypasch E, Williams JI, Wood-Dauphinee S (2005). Gastrointestinal Quality of Life Index: development, validation and application of a new instrument. Br J Surg.

[13] Sandblom GMDP, Videhult PMD, Karlson B-MMDP (2009). Validation of gastrointestinal quality of life index in Swedish for assessing the impact of gallstones on health-related quality of life. Value Health.

[14] Posegger KR, Maeda CT, Taveira JP (2022). Brazilian-Portuguese validation assessment of the Gastrointestinal Quality of Life Index for patients after laparoendoscopic cholecystectomy. J Laparoendosc Adv Surg Tech A.

[15] Meijnikman AS, Aydin O, Prodan A (2020). Distinct differences in gut microbial composition and functional potential from lean to morbidly obese subjects. J Intern Med.

[16] Meijnikman AS, Davids M, Herrema H (2022). Microbiome-derived ethanol in nonalcoholic fatty liver disease. Nat Med.

[17] Costea PI, Zeller G, Sunagawa S (2017). Towards standards for human fecal sample processing in metagenomic studies. Nat Biotechnol.

[18] Wood DE, Salzberg SL (2014). Kraken: ultrafast metagenomic sequence classification using exact alignments. Genome Biol.

[19] Wood DE, Lu J, Langmead B (2019). Improved metagenomic analysis with Kraken 2. Genome Biol.

[20] Almeida A, Nayfach S, Boland M (2021). A unified catalog of 204,938 reference genomes from the human gut microbiome. Nat Biotechnol.

[21] Lu J, Breitwieser FP, Thielen P (2017). Bracken: estimating species abundance in metagenomics data. PeerJ Computer science.

[22] McMurdie PJ, Holmes S (2013). Phyloseq: An R package for reproducible interactive analysis and graphics of microbiome census data. PloS One.

[23] Oksanen J, Blanchet FG, Friendly M (2017). Vegan: Community Ecology Package. R package version 1.

[24] Kaci G, Goudercourt D, Dennin V (2014). Anti-inflammatory properties of Streptococcus salivarius, a commensal bacterium of the oral cavity and digestive tract. Appl Environ Microbiol.

[25] Nicolas GG, Lavoie MC (2011). Streptococcus mutans and oral streptococci in dental plaque. Can J Microbiol.

[26] Stefura T, Zapała B, Gosiewski T (2022). Changes in the composition of oral and intestinal microbiota after sleeve gastrectomy and Roux-En-Y gastric bypass and their impact on outcomes of bariatric surgery. Obes Surg.

[27] Porat D, Vaynshtein J, Gibori R (2021). Stomach pH before vs. after different bariatric surgery procedures: clinical implications for drug delivery. Eur J Pharm Biopharm.

[28] Stefura T, Zapala B, Stoj A (2020). Does postoperative oral and intestinal microbiota correlate with the weight-loss following bariatric surgery?-A cohort study. J Clin Med.

[29] Louis P, Flint HJ (2017). Formation of propionate and butyrate by the human colonic microbiota. Environ Microbiol.

[30] Tang G, Du Y, Guan H (2022). Butyrate ameliorates skeletal muscle atrophy in diabetic nephropathy by enhancing gut barrier function and FFA2-mediated PI3K/Akt/mTOR signals. Br J Pharmacol.

[31] Petrov VA, Saltykova IV, Zhukova IA (2017). Analysis of gut microbiota in patients with Parkinson’s disease. Bull Exp Biol Med.

[32] de Goffau MC, Luopajarvi K, Knip M (2013). Fecal microbiota composition differs between children with beta-cell autoimmunity and those without. Diabetes.

[33] Robert C, Chassard C, Lawson PA (2007). Bacteroides cellulosilyticus sp. nov., a cellulolytic bacterium from the human gut microbial community. Int J Syst Evol Microbiol.

[34] Ulker İ, Yildiran H (2019). The effects of bariatric surgery on gut microbiota in patients with obesity: a review of the literature. Biosci Microbiota Food Health.

[35] Ghosh TS, Rampelli S, Jeffery IB (2020). Mediterranean diet intervention alters the gut microbiome in older people reducing frailty and improving health status: the NU-AGE 1-year dietary intervention across five European countries. Gut..

[36] Wang Z, Zhong J, Meng X, Gao J (2021). The gut microbiome-immune axis as a target for nutrition-mediated modulation of food allergy. Trends Food Sci Technol.

[37] Palmisano S, Campisciano G, Silvestri M (2020). Changes in gut microbiota composition after bariatric surgery: a new balance to decode. J Gastrointest Surg.

[38] Montravers P, Lepape A, Dubreuil L (2009). Clinical and microbiological profiles of community-acquired and nosocomial intra-abdominal infections: results of the French prospective, observational EBIIA study. J Antimicrob Chemother.

[39] Nagao-Kitamoto H, Kamada N (2017). Host-microbial cross-talk in inflammatory bowel disease. Immune network.

[40] Benus RFJ, van der Werf TS, Welling GW (2010). Association between Faecalibacterium prausnitzii and dietary fibre in colonic fermentation in healthy human subjects. Br J Nutr.

[41] Haderstorfer B, Psycholgin D, Whitehead WE (1989). Intestinal gas production from bacterial fermentation of undigested carbohydrate in irritable bowel syndrome. Am J Gastroenterol.

[42] Fernandes R, Beserra BTS, Mocellin MC (2016). Effects of prebiotic and synbiotic supplementation on inflammatory markers and anthropometric indices after Roux-en-Y gastric bypass: a randomized, triple-blind, placebo-controlled pilot study. J Clin Gastroenterol.

[43] Durazzi F, Sala C, Castellani G (2021). Comparison between 16S rRNA and shotgun sequencing data for the taxonomic characterization of the gut microbiota. Sci Rep.

