# Supplementary figures and images for: Identifying Gut Microbiota associated with Gastrointestinal Symptoms upon Roux-en-Y Gastric Bypass
Source: Obes Surg. 2023 Apr 24;33(6):1635–45. doi: 10.1007/s11695-023-06610-6 (PMC10235151; doi:10.1007/s11695-023-06610-6)

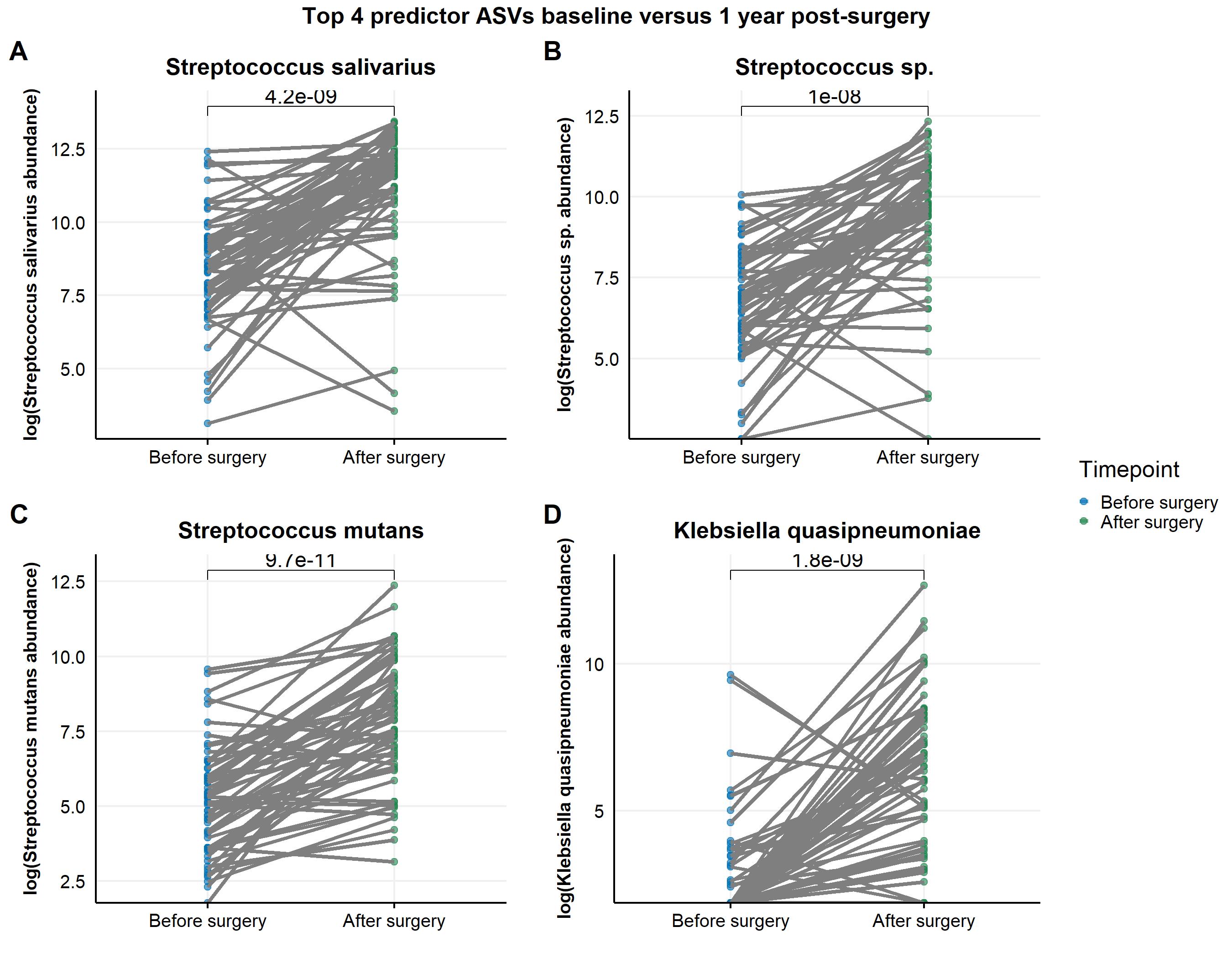

Supplement: Supplementary file 1 — (JPG 453 kb) [file 11695_2023_6610_MOESM1_ESM.jpg]

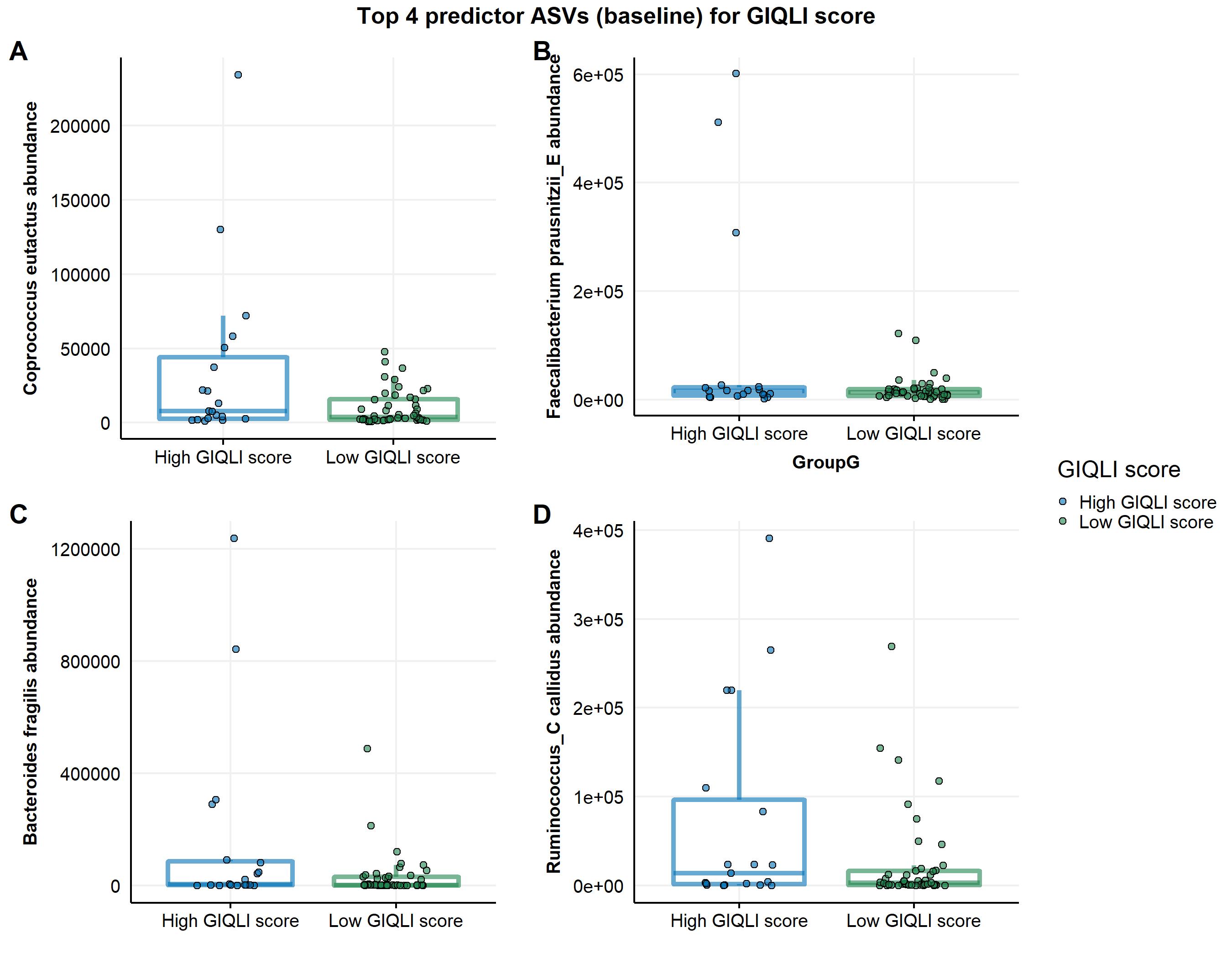

Supplement: Supplementary file 2 — (JPG 301 kb) [file 11695_2023_6610_MOESM2_ESM.jpg]

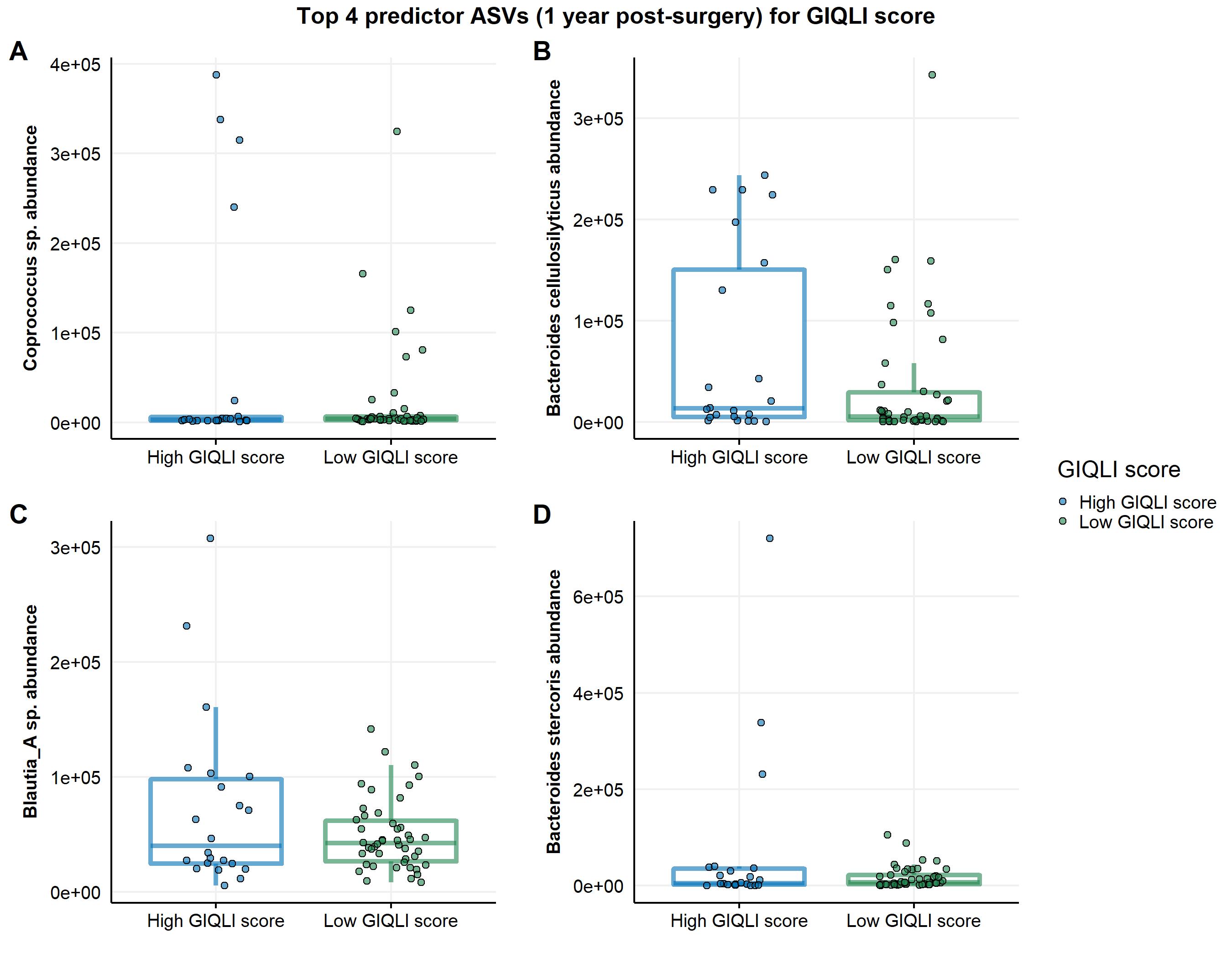

Supplement: Supplementary file 3 — (JPG 301 kb) [file 11695_2023_6610_MOESM3_ESM.jpg]

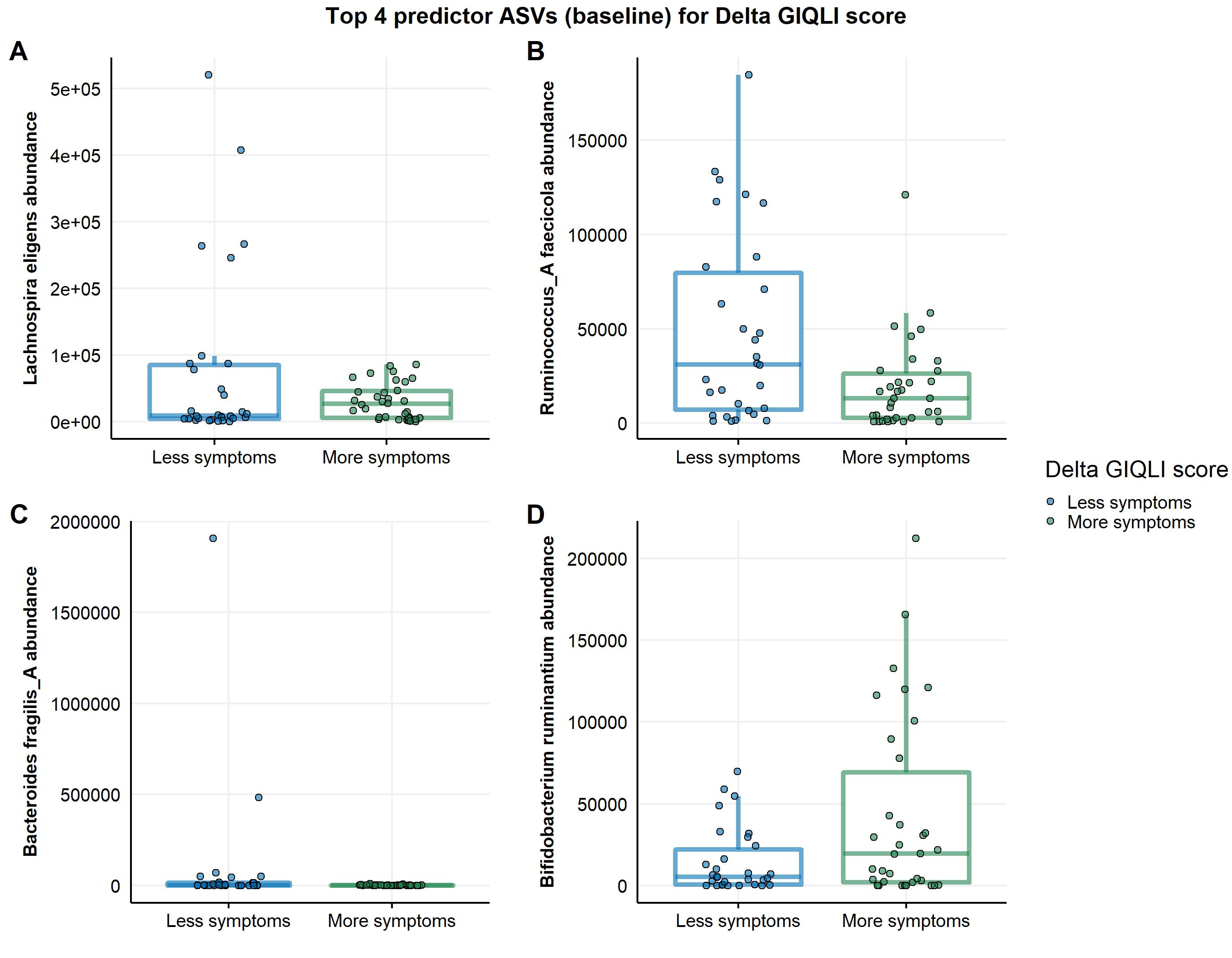

Supplement: Supplementary file 4 — (JPG 310 kb) [file 11695_2023_6610_MOESM4_ESM.jpg]

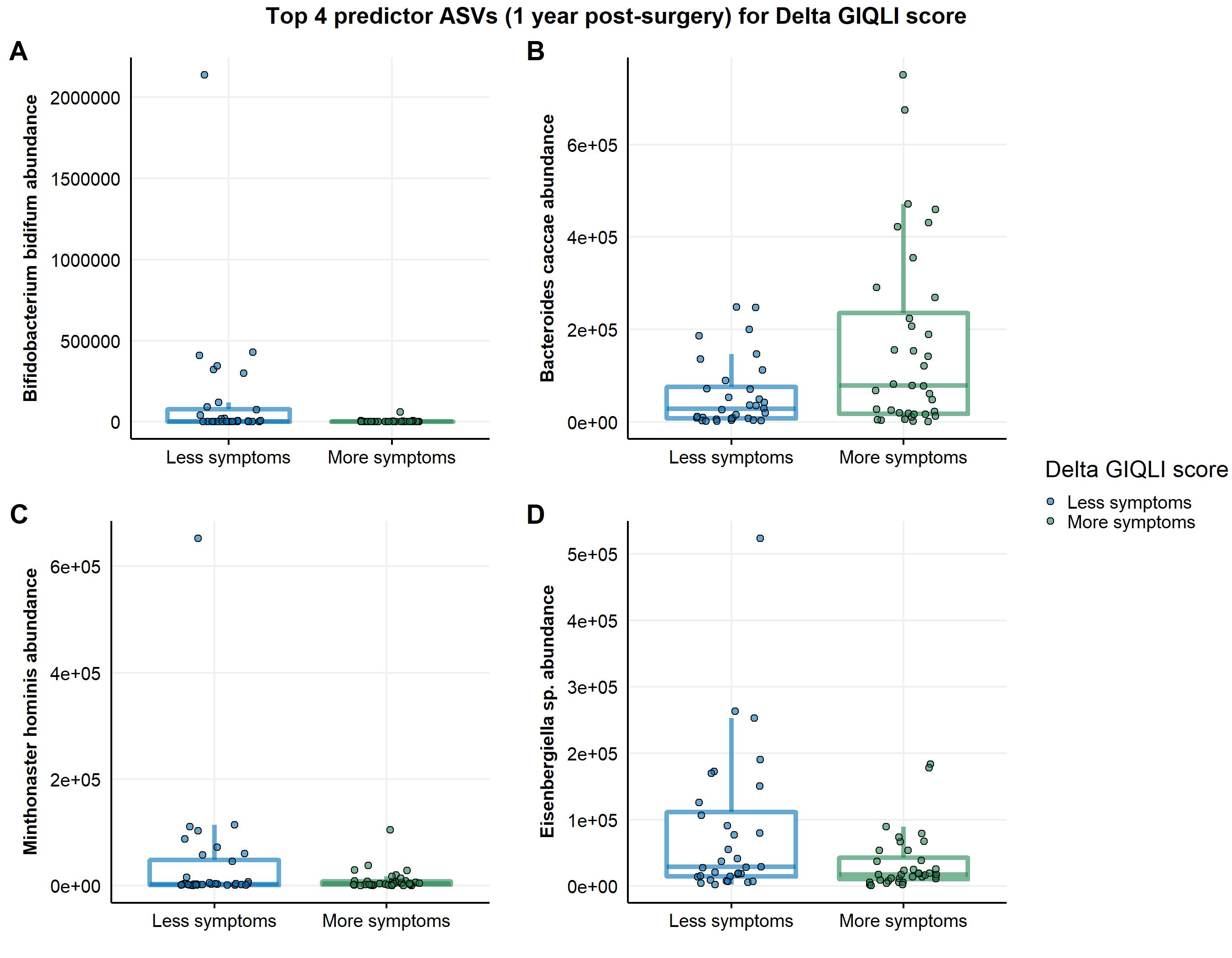

Supplement: Supplementary file 5 — (JPG 305 kb) [file 11695_2023_6610_MOESM5_ESM.jpg]
